# Profiling Cancer-Associated Fibroblasts in Melanoma

**DOI:** 10.3390/ijms22147255

**Published:** 2021-07-06

**Authors:** Federica Papaccio, Daniela Kovacs, Barbara Bellei, Silvia Caputo, Emilia Migliano, Carlo Cota, Mauro Picardo

**Affiliations:** 1 Laboratory of Cutaneous Physiopathology and Integrated Center of Metabolomics Research, San Gallicano Dermatological Institute, IRCCS, 00144 Rome, Italy; daniela.kovacs@ifo.gov.it (D.K.); barbara.bellei@ifo.gov.it (B.B.); silvia.caputo@ifo.gov.it (S.C.); mauro.picardo@ifo.gov.it (M.P.); 2 Department of Plastic and Regenerative Surgery, San Gallicano Dermatological Institute, IRCCS, 00144 Rome, Italy; emilia.migliano@ifo.gov.it; 3 Genetic Research, Molecular Biology and Dermatopathology Unit, San Gallicano Dermatological Institute IRCCS, 00144 Rome, Italy; carlo.cota@ifo.gov.it

**Keywords:** melanoma, CAF, tumor microenvironment, stroma, inflammation

## Abstract

Solid tumors are complex systems characterized by dynamic interactions between neoplastic cells, non-tumoral cells, and extracellular components. Among all the stromal cells that populate tumor microenvironment, fibroblasts are the most abundant elements and are critically involved in disease progression. Cancer-associated fibroblasts (CAFs) have pleiotropic functions in tumor growth and extracellular matrix remodeling implicated in local invasion and distant metastasis. CAFs additionally participate in the inflammatory response of the tumor site by releasing a variety of chemokines and cytokines. It is becoming clear that understanding the dynamic, mutual melanoma–fibroblast relationship would enable treatment options to be amplified. To better characterize melanoma-associated fibroblasts, here we analyzed low-passage primary CAFs derived from advanced-stage primary skin melanomas, focusing on the immuno-phenotype. Furthermore, we assessed the expression of several CAF markers and the production of growth factors. To deepen the study of CAF–melanoma cell crosstalk, we employed CAF-derived supernatants and trans-well co-culture systems to evaluate the influences of CAFs on (i) the motogenic ability of melanoma cells, (ii) the chemotherapy-induced cytotoxicity, and (iii) the release of mediators active in modulating tumor growth and spread.

## 1. Introduction

Melanoma is an aggressive melanocytic neoplasm with an increasing annual number of cases, more so than other solid tumors, and treatment resistant [1,2,3]. Despite accounting for about 4% of skin cancers, melanoma is responsible for the majority of cutaneous cancer mortality and about 1–2% of all cancer deaths [4,5]. The survival of patients is closely associated with early detection. However, little is understood about predicting biomarkers to identify a prognostic subtype of melanoma, including the specific contribution of the tumor microenvironment. Although a tumor is a complex system resulting from the interactions between transformed cells and the surrounding tissue, studies of oncogenesis have mainly focused on the former [6], particularly in melanoma due to the highest mutational burden of any cancer, partially attributed to UV-induced DNA damage. However, recent studies have shown that stromal cells surrounding the cancer nest play a major role in progression and invasion [7]. Activated fibroblast, called cancer-associated fibroblast (CAF), is a recurrent cell type present in the tumor microenvironment [8,9]. This type of fibroblast demonstrated a distinct phenotype compared to its “normal” counterpart [10]. Starting from the early tumor stage, due to continuous paracrine stimulation by transformed cells, enclosing stromal fibroblasts are induced to initiate phenotypic, molecular, and biochemical transitions and to transdifferentiate into CAFs. These cells are frequently described as similar to myofibroblasts abundantly expressing the highly contractile protein α-smooth muscle actin (α-SMA) seen during the wound healing process and in fibrotic conditions [11]. CAFs are distinguished from their normal counterparts by the expression of several markers, such as fibroblast-specific protein-1 (FSP-1, also referred to as S100A4), fibroblast-activating protein (FAP), platelet-derived growth factor receptor-alpha/beta (PDGFR α/β), tenascin-C, desmin, collagen 11-α1 (COL11A1), vimentin, and fibronectin. However, a univocal molecular definition of a CAF profile is as yet lacking. Several studies have revealed CAF heterogeneity, which implies cancer type-specific features, inter-patient and stage-linked variability, and intra-tumoral subsets [12,13,14]. A specific profile of fibroblast-associated melanoma genesis has not yet been defined. On the other hand, chronic and long-term exposure to UV radiation, the major etiological factor in skin cancer, causes changes in dermal fibroblasts, resulting in increased secretion of growth factors and in the acquisition of the senescent-like phenotype similar to that observed in cancer-activated ones [15,16,17]. Therefore, modification in the stroma can act independently of melanocytic cell alterations, thus moving as a driver of the tumorigenic process. Consequently, in the skin, activation of fibroblasts might precede the recruitment of stromal cells by tumoral cells, a process that includes paracrine stimulation due to the pro-mitogenic tumor milieu, considering also the extraordinary secretory activity of melanoma cells [18,19]. The presence of a large number of CAFs in the tumor stroma is associated with an increased risk of invasion, metastasis, and poor prognosis of several different types of cancers [20,21,22]. Because of the great capacity of fibroblasts to secrete extracellular matrix (ECM) components and ECM-remodeling enzymes, both CAF expression profile and CAF density drive the architecture modification of host tissue. In melanoma, the stiffness of the surrounding ECM has been linked to tumor aggressiveness and the acquisition of drug resistance [1].

The capacity to sustain cancer depends largely on augmented pro-mitogenic peptides that facilitate tumor growth, whereas secreted inflammatory mediators operate in conflicting modalities: both tumor-antagonizing and tumor-promoting [23,24]. The balance between pro-inflammatory and anti-inflammatory cytokines in the tumor area strongly impacts a patient’s prognosis. Melanoma-associated fibroblasts have also been demonstrated to play an important role in the induction of immune suppression via melanoma–stroma crosstalk, and several studies have related CAF abundance with targeted drug resistance as well as checkpoint inhibitor resistance in advanced melanoma [17,25,26,27]. Among the immune modulators involved in CAF activation, IL-1β seems to be a driver of melanoma invasion in vitro and in vivo [28]. Cytokines produced by dermal fibroblasts, such as interleukin-6 and -8 (IL-6, IL-8), interferon-γ (INF-γ), tumor necrosis factor-alpha (TNF-α) [29,30], and a variety of C-X-C motif chemokine ligands (CXCLs) [30], can mobilize immune cells. 

In this study, we aimed to characterize a panel of cancer-associated fibroblast cell lines freshly isolated from melanoma lesions, focusing on their phenotypical and functional features. In addition, the influences of CAFs on melanoma cell behavior were investigated by employing cell supernatants and co-culture systems to mimic in vitro the cell–cell crosstalk existing in the tumor microenvironment.

## 2. Results

### 2.1. Expression Profile of Melanoma-Associated Fibroblast Markers

**Figure 1:**
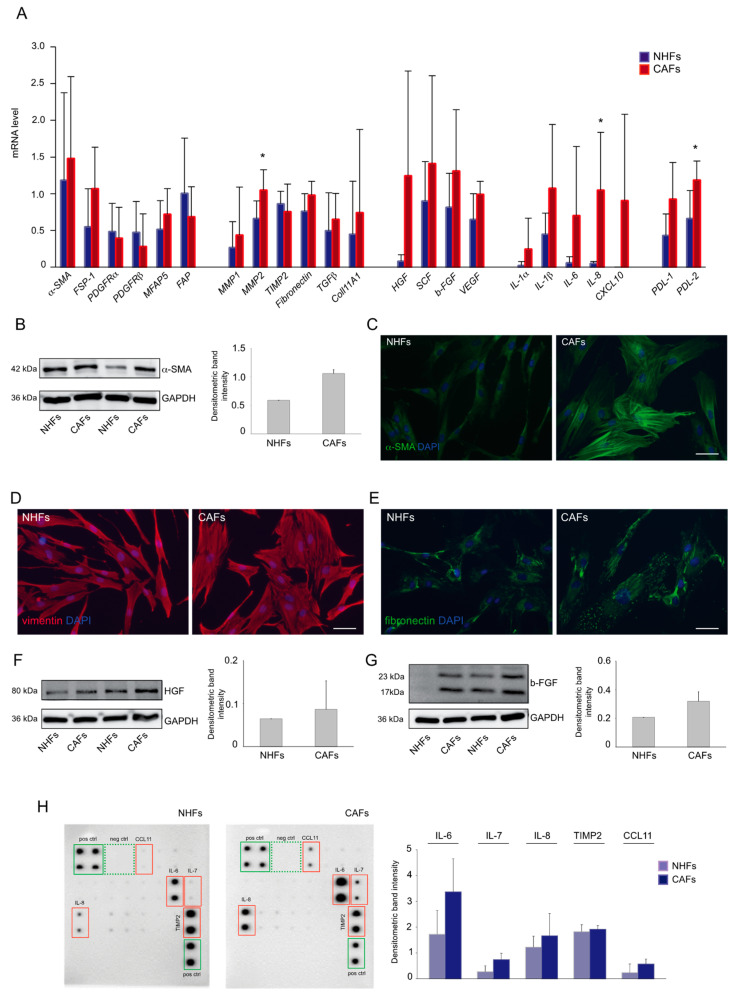
Characterization of melanoma CAF cultures. (**A**) mRNA levels of a panel of markers commonly used to identify cancer-associated fibroblasts. β-actin mRNA was used as reference. (NHFs *n* = 6, CAFs *n* = 7) * *p* < 0.05 vs. NHFs. (**B**) Western blot and densitometric analysis of α-SMA expression in NHFs (*n* = 6) and CAFs (*n* = 7). (**C**) Immunofluorescence analysis of α-SMA (green) expression detected in control (*n* = 6) and cancer-associated fibroblasts (*n* = 7). Nuclei are counterstained in DAPI. Scale bar: 50 µm. Immunofluorescence staining for vimentin (red) (**D**) (NHFs = 6; CAFs = 7) and fibronectin (green) (**E**) (NHFs = 5; CAFs = 5). Nuclei are counterstained in DAPI. Scale bar: 50 µm. Western blot and densitometric analyses of HGF (**F**) and b-FGF (**G**) in control (*n* = 6) and cancer-associated fibroblasts (*n* = 7). Protein array and densitometric analysis (**H**) showing the expression of inflammatory-related markers in NHFs (*n* = 6) and CAFs (*n* = 7).

We firstly characterized primary fibroblast cultures from fresh skin melanoma samples, evaluating the mRNA level of a large panel of markers commonly used to identify CAFs in various tumor types. We selected groups of genes covering biological processes linked to tumor growth and invasion and known to involve CAFs, i.e., inflammation, tissue remodeling, angiogenesis, and metabolic reprogramming. In addition, as numerous studies have recently revealed a role for CAFs in mediating anti-tumor immune response [31], we evaluated the expression of PD ligand 1 and 2 (PDL-1, PDL-2). The levels of most of the analyzed genes tended to be higher in CAFs in comparison to NHFs. Among them, MMP2, IL-8, and PDL-2 reached statistical significance. The high variability in basal level of gene expression may reflect the wide heterogeneity and complexity of the tumor milieu existing in vivo. Expression levels of the extensively identified CAF marker α-SMA was increased, as well as that of FSP1, whereas other commonly used markers such as PDGFRα/β did not show differences in their mRNA levels between CAFs and NHFs. No evident differences resulted in the mRNA transcripts for the CAF-associated protein FAP. A relatively high level of extracellular matrix deposition- and remodeling-related genes fibronectin, MMP1, and MMP2 was detected. We also observed an increased mRNA level of Coll11A1, whose expression has been referred to as specific for CAFs and has been associated with cancer progression and poor survival in several malignancies, including metastatic melanoma [32,33]. Increased levels of secreted factors involved in tumor growth and progression were detected. In particular, in comparison to NHFs, CAFs expressed a higher amount of hepatocyte growth factor (HGF), stem cell factor (SCF), basic fibroblast growth factor (b-FGF), and vascular endothelial growth factor (VEGF), which are growth factors with pro-mitogenic and -motogenic effects favoring local and distant invasion and angiogenesis. Among the inflammatory mediators, cytokines IL-1α, IL-1β, IL-6, and IL-8 and chemokine CXCL10 were highly expressed, suggesting a function of CAFs in tumor-mediating inflammation [34] (Figure 1A). The analysis of several markers at the protein level confirmed the overall tendency of an increased expression in CAFs in comparison to controls. In particular, CAF cultures showed a high expression of α-SMA in comparison to normal fibroblasts, as assessed by Western blot (Figure 1B). Parallel immunofluorescence analysis demonstrated its staining pattern as cytoplasmic positive stress fibers extended along with the cell (Figure 1C). All CAF cultures displayed a strong expression of vimentin and fibronectin, which was distributed throughout the cytoplasm (Figure 1D,E). Similarly, the expression level of b-FGF and HGF was higher in several CAFs compared to control cells (Figure 1F,G). CAFs are believed to be an important source of chemokines, including C-C motif chemokine ligand (CCL) and CXCLs [35]. As the mRNA analyses revealed the induction of several inflammatory genes, we deepened the characterization of the inflammatory profile of CAFs using a human cytokine protein array. The results demonstrated a higher expression of IL-6 and IL-8 together with IL-7, CCL11, and tissue inhibitor of metalloproteinase 2 (TIMP2) (Figure 1H). However, contrary to gene expression data, IL-1α and IL-1β were undetectable.

### 2.2. CAF Influence on Cancer Cell Migration

**Figure 2:**
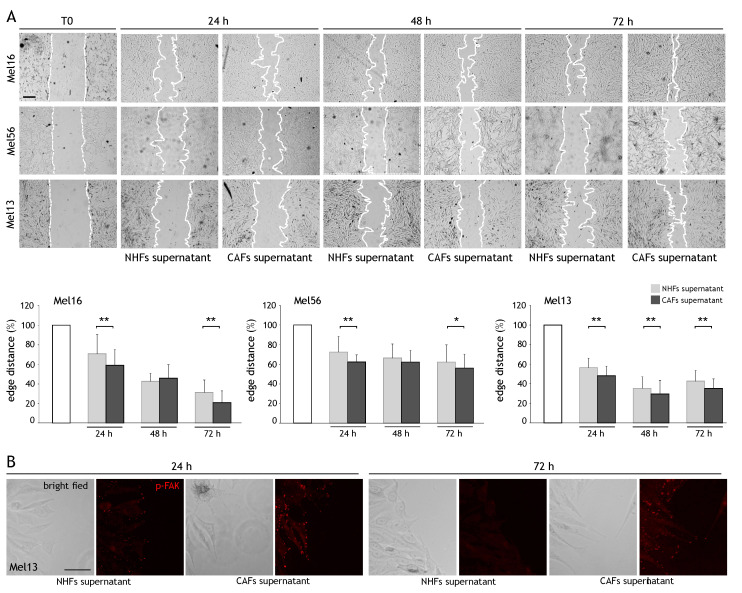
Effects of CAF supernatant on melanoma cell migration. (**A**) Representative images of the scratch wound assay of melanoma cells maintained in NHF and CAF supernatant at T0, 24, 48, and 72 h and quantitative analysis of the distance of the leading edge. Melanoma cell lines (*n* = 3) were treated with the supernatant obtained from 1 sample of NHFs and 1 sample of CAFs. For each time point evaluated, the reduction of edge distance of melanoma cells treated with NHF supernatant was compared with that of the cells maintained with CAF supernatant. Scale bar: 200 µm. * *p* < 0.05 and ** *p* < 0.01 vs. NHF supernatant-treated cells. (**B**) Bright-field and immunofluorescence analysis of pFAK expression (red) in Mel13 melanoma cells grown in NHF (*n* = 2) and CAF (*n* = 2) supernatant for the indicated time points. Scale bar: 50 µm.

As CAFs release several mediators known to promote tumor cell motility, thus favoring invasion and metastasis, we investigated whether CAFs affect melanoma cell migration. We performed the wound scratch assay on primary cultures of freshly isolated melanoma cells and assessed the effects of the supernatant obtained from CAFs and NHFs on their migratory potential. We observed a reduction in the leading edge distance after all time points evaluated in both NHF and CAF supernatant-treated cells. However, comparing the effect of control- and CAF-conditioned medium, a faster reduction in the scratched area was measured in melanoma cells maintained in CAF supernatant (Figure 2A). Based on these results, we next evaluated the phosphorylation of the focal adhesion kinase (FAK), as the activation of FAK and its related signaling pathways are known to stimulate cell migration [36]. Immunolabeling for FAK^tyr397^ on wounded Mel13 cells revealed positive reactivity toward the margins of the scratched areas in cells maintained with both CAF and NHF supernatants after 24 h. Interestingly, a higher expression of pFAK was conserved up to 72 h in melanoma cells in the presence of CAF-derived medium in comparison to NHF-conditioned medium, suggesting a contribution of CAF-secreted factors in promoting the persistence of FAK activation and consequently of cell motility (Figure 2B).

### 2.3. CAF-Secreted Factors Protect Melanoma Cells from Acute Toxicity of Chemotherapy

**Figure 3:**
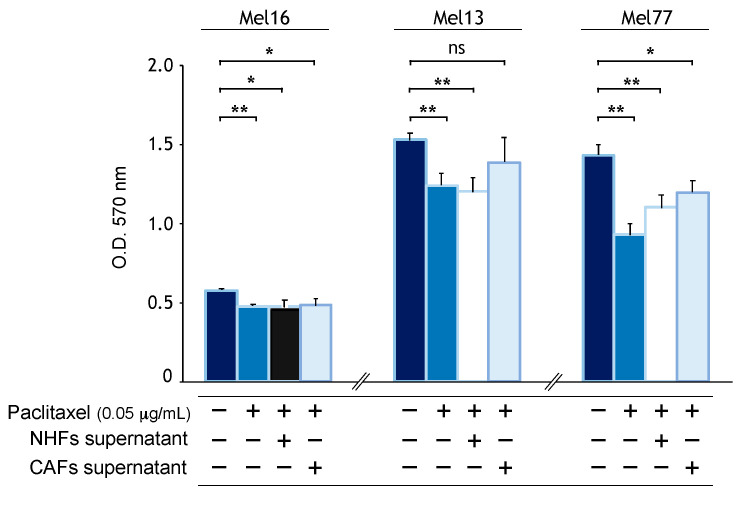
Effects of CAF supernatant on chemotherapy-induced cytotoxicity. MTT assay of melanoma cells treated with paclitaxel (0.05 µg/mL) in the presence or absence of NHF and CAF supernatant. Histograms show mean ± SD of three independent experiments performed with three different melanoma cell lines (Mel13, Mel77, and Mel16) treated with *n* = 3, *n* = 4, and *n* = 4 NHF supernatants, respectively, or with *n* = 4, *n* = 5, and *n* = 5 CAF supernatants, respectively. * *p* < 0.05 and ** *p* < 0.01 vs. control untreated cells. ns: not significant.

Next, we investigated the effect of CAF-secreted factors in anti-tumor-induced cytotoxicity. For this purpose, we used paclitaxel (PTX), a microtubule-targeting drug that alters the normal polymerization/depolymerization process during mitosis. Consistent with paclitaxel’s mechanism of action involving interference with the normal breakdown of microtubules during cell division, rapidly dividing tumor cells underwent apoptotic cell death and MTT assay evidenced a significant decrease in metabolic activity. In the presence of fibroblast supernatants, melanoma cells became partially protected. A tendency for higher protection was observed in the case of CAFs compared to NHFs in Mel13 and Mel77 but not in Mel16 (Figure 3). Similar results were observed with cisplatin, another antitumor drug acting with a different mechanism of action (data not shown), confirming the existence of pro-survival factors released by CAFs.

### 2.4. Melanoma Cells Influence the Paracrine Activity of CAFs

**Figure 4:**
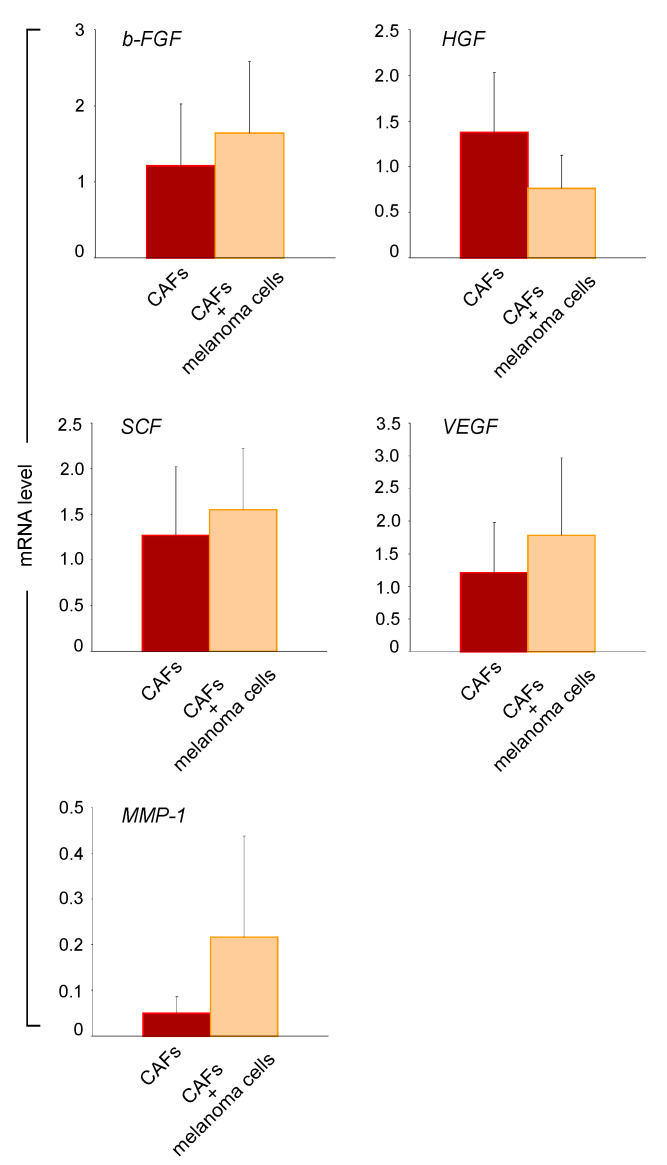
Gene expression evaluation in CAF/melanoma co-culture. An RT-PCR study was carried out to compare CAF gene expression in monocultured (*n* = 3) and co-cultured (CAFs *n* = 3, melanoma cell lines *n* = 3) conditions. Histograms show the modification of b-FGF, HGF, SCF, VEGF, and MMP1 gene expression. β-actin mRNA was used as reference. Results are the mean ± SD of at least three independent experiments.

To better understand the paracrine interactions between melanoma cells and CAFs, we performed co-culture experiments using a trans-well system and compared the gene expression profile of a panel of secreted mediators in CAF/melanoma co-cultures with those of CAF monoculture. This analysis showed a partial increase in b-FGF, SCF, and VEGF gene expression (Figure 4). A mild decrease in HGF expression was detected, whereas the transcription level of MMP1 was upregulated. Co-culturing CAFs with melanoma cells tended to empower growth factors and MMP1 production, suggesting a reciprocal dependency between CAFs and cancer cells.

## 3. Discussion

In recent years, the stromal compartment has emerged to actively contribute to tumor progression, with pleiotropic effects via paracrine and architectural interactions with transformed cells. The tumor milieu is highly complex per se due to the co-existence of different cell types beyond malignant cells, i.e., cancer-associated fibroblasts and endothelial and immune cells. Such complexity is further heightened by the concurrent existence of several subpopulations of CAFs, whose phenotype shows differences among tumors of the same type in different patients and inside the stroma of the same tumor. Modification of the microenvironment is primarily a response to the parenchymal injury associated with neoplastic onset. However, several external factors might contribute to this change, especially in the skin, because of the chronic and long-term exposure to UV radiation. Consequently, unlike internal organs, dermal fibroblasts undergo time- and lifestyle-dependent changes that can act in a pro-tumorigenic fashion independently of tumor inception. These include the expression of several intracellular and membrane surface-bound markers that have been only in part linked to a clear functional role in oncogenesis and the production of a variety of soluble factors involved in paracrine signaling. Nevertheless, due to the lack of specific markers, a precise description of melanoma-associated fibroblasts remains undefined and partially overlapped with that characterizing a senescent-like phenotype. This study attempted to investigate CAF profile in skin melanoma matching low-passage stromal fibroblast cultures with age-matched normal fibroblasts, focusing on relevant pro-tumorigenic functions of mesenchymal cells. The analysis of previously described phenotypical characteristics of CAFs, such as the expression of α-SMA, FSP-1, and PDGFR α and β, partially confirmed a typical cancer-associated layout. A high level of α-SMA in CAFs confirmed a fibroblast activated state and the presence of a fibrotic-like response. However, α-SMA-positive CAFs critically contribute to tumor progression when producing chemokines and cytokines [37]. On the other hand, FAP expression appeared similar to healthy fibroblasts at both the mRNA and protein levels. In contradiction to the previous report [38], we failed to demonstrate the overexpression of PDGFRs. However, the reliability of these surface markers in the context of tumors is still debated since they are generally considered fibroblast markers abundantly present in healthy counterparts as well. Numerous previous studies have highlighted a pro-mitogenic feature of CAFs due to the ability to synthesize and secrete a large variety of pro-growing peptides [39]. Herein, comparative gene expression profiling of normal tissue-derived fibroblasts and CAFs demonstrated the overexpression of a variety of growth and pro-angiogenic factors such as b-FGF, HGF, SCF, and VEGF, confirming the pro-mitogenic attitude of melanoma-associated fibroblasts. Moreover, amplification of growth factors produced in the co-culture model underlined the capacity of melanoma cells to redirect gene expression in fibroblasts, whereas the reciprocal effect driven by stromal cells modestly emerged (data not shown). The tumor-supportive role exerted by stromal fibroblasts is also evidenced by the protection of melanoma cells exposed to the chemotherapeutic agent paclitaxel. The paracrine activity of CAFs acts not only directly on melanoma cells but also through the modulation of local inflammation [40]. In this study, the immune-regulatory ability of melanoma CAFs was demonstrated by the ample production of several cytokines and chemokines, including IL-1α, IL-1β, IL-6, IL-8, and CXCL10. An interesting but still controversial point was recently raised based on the observation that CAFs from melanoma might express programmed death ligand-1 and/or -2 [41]. In agreement with previous findings, we confirmed overexpression of both PD-L1 and PD-L2 in melanoma-associated fibroblasts. Recently, it has been reported that CAFs promote therapeutic resistance mainly through the secretion of multiple cytokines and chemokines. Liu et al. indicated the immunosuppressive effects of IL-6 in hepatocellular carcinoma cancer (HCC). Moreover, the ability of IL-6 blockade to overcome anti-PD-L1 resistance has been demonstrated in an HCC tumor model [42]. Yet, other studies have revealed that CAF-derived IL-8 was associated with gastric cancer chemoresistance [43]. Similarly, specific CAF subtypes associated with immunotherapy resistance in melanoma have been identified [44,45]. These observations support the tumor microenvironment as a novel therapeutic target. To functionally analyze the mutual interactions of CAFs and melanoma cells, we next examined the effects of CAF-derived supernatants on the migration ability of melanoma cells. In line with previous reports [28], we observed higher motility of melanoma cells when cultured with CAF supernatants in comparison to those from normal fibroblasts. Such an effect was also accompanied by the longer persistence of phosphorylated FAK, whose overexpression and/or constitutive activity have been linked to melanomas with higher metastatic and aggressive potential [36].

These results suggest a contribution of CAFs in promoting melanoma invasiveness via the secretion of factors, which may act both alone and in synergic combination. Among known pro-invasive mediators, we observed a positive expression and release of a conspicuous amount of IL-6 and IL-8 cytokines by melanoma CAFs, which may corroborate previously reported data showing a link between this inflammatory cytokine and melanoma invasiveness [30]. In addition, several metalloproteinases, included in the class of proteases, may be possible active players involved in stimulating melanoma mobility. Matrix degradation mediated by these enzymes has been shown to play an indirect role during tumor invasion [22]. Accordingly, our results reveal the upregulation of the MMP1 gene in CAF cultures that is further exacerbated by direct CAF–melanoma cell co-cultures. As reported for other malignancies, the wide variability observed in the expression of several markers may be justified by the simultaneous presence of heterogeneous subsets of CAF also in the melanoma microenvironment. Distinct subpopulations might stimulate different aspects during malignancy. In several tumors, CAF heterogeneity implicates the presence of at least two distinctive CAF types: the myofibroblast-like sub-population operating mainly on tissue remodeling and the inflammatory one responsible for immune system modulation [45]. Here, overexpression of α-SMA, IL-6, and IL-8 is the most prominent characteristic of melanoma-associated fibroblasts, suggesting the possible concomitant expression of these markers. On the other hand, the CAF feature partially overlaps with the hypersecretory phenotype of senescent cells [15]. Of note, the senescence-associated secretory phenotype (SASP) is characterized by an increased release of diverse pro-inflammatory cytokines, including a consistent amount of IL-6 and IL-8 [46,47,48,49]. Thus, in the context of skin tumors, chronic exposure to extrinsic damage, accelerating the aging process, may lead to neoplastic predisposition independently of cancer onset and/or of strengthening the CAF phenotype. Accordingly, we previously demonstrated that in hyperpigmentary disorders such as solar lentigo, photo-damaged fibroblasts produce increased levels of growth factors [50]. Defining the features of fibroblasts able to favor melanoma development even earlier in its onset is challenging for melanoma prevention, whereas the identification of characteristics induced by tumor cells is a focal point of anti-stromal therapies. Although this study has some limitations, including the restricted sample size used and the in vitro model, which may not effectively mirror the in vivo complexity, our results documented an overall tumor-supporting phenotype of melanoma-associated fibroblasts that persists in in vitro monoculture condition. In addition, dynamic bidirectional crosstalk operating in the co-culture system evidenced that melanoma cells might rapidly influence gene expression in stromal cells.

## 4. Methods

### 4.1. Ethic Statement

The Declaration of Helsinki Principles was followed and patients gave written informed consent to collect samples of human material for research. Furthermore, the Institutional Research Ethics Committee (Istituti Regina Elena and San Gallicano) approved all research activities involving human subjects (For healthy subjects: Prot CE/286/06, approved on 21 April 2006; for melanoma patients: Prot: 1174/19, approved on 29 January 2019).

### 4.2. Cell Cultures and Treatments

Six primary cultures of normal human fibroblasts (NHFs) were isolated from human skin fragments obtained from plastic surgery. Briefly, the skin was cut into approximately 4 mm^2^-sized pieces and incubated overnight at 4 °C with dispase (2.5 mg/mL) to separate the epidermis from the dermis. The dermis was digested with collagenase 0.35% for 1 h at 37 °C and the obtained NHFs were maintained in culture with DMEM (EuroClone S.p.A., Milan Italy) supplemented with 10% fetal bovine serum (FBS) and antibiotics (Hyclone Laboratories, South Logan, UT, USA). Cancer-associated fibroblasts (CAFs) were isolated from eight melanoma tumor samples using the same method. Melanoma cells were isolated exclusively from excess parts of the biopsy collected for histological examinations without compromising the standard diagnostic procedure. The tissue was manually crumbled into small pieces and then incubated with collagenase 0.35% for 45 min at 37 °C, centrifuged, resuspended, and grown in OptiMEM (Life Technologies, Invitrogen, Milan, Italy) medium containing 10% FBS and antibiotics. All the experiments were performed at low cell culture passages (2–12). Cell types and melanoma patient characteristics have been reported in Supplementary Table S1.

NHF and CAF supernatants were collected after 96 h from cell cultures maintained in DMEM plus antibiotics either in the presence or in the absence of FBS and then stored at −80 °C. For protein array experiments, FBS was omitted.

For paclitaxel (Sigma-Aldrich S.r.l., Milan, Italy) treatment, 2.5 × 10^4^ melanoma cells were seeded into 24-well plates for 24 h to adhere. Then, the growth medium was changed with a mixture of a half fresh medium and half CAF- or NHF-conditioned medium containing 0.05 µg/mL paclitaxel (or not for control cells). At the experimental endpoint (72 h), cells were incubated with 3-(4,5 dimethylthiazol)-2,5-diphenyl tetrazolium bromide (MTT) for 2 h. After this time, the medium was removed and the resulting crystals were solubilized in DMSO. The absorbance was measured at 570 nm with a reference wavelength of 690 nm. Absorbance readings were subtracted from the value of the blank wells. The experiments were performed in duplicate.

### 4.3. Transwell Co-Culture

Briefly, 6 mm transwells with 3.0 μm pore membrane insert were used with CAFs and cancers cells in the lower and upper compartment, respectively. A total of 5.0 × 10^4^ CAFs per well were seeded in the lower compartment and 7.5 × 10^4^ melanoma cells were seeded in the upper compartment and incubated at 37 °C 5% CO_2_ overnight. After 24 h, fresh OptiMEM + 10% FBS was replaced and transwell inserts containing melanoma cells were added to CAFs seeded in the lower compartment. After 72 h, RNA was extracted and analyzed.

### 4.4. Western Blot Analysis

Cell extracts were prepared with RIPA buffer containing protease and phosphatase inhibitors. Proteins were separated on SDS-polyacrylamide gels, transferred to nitrocellulose membranes, and then treated with the following primary antibodies: anti-b-FGF (1:500) (Upstate Biotechnology, Inc., Lake Placid, NY, USA), anti-HGF (1:1000) (AbCam, Cambridge, UK and anti-αSMA (1:1000) (Sigma Aldrich, Merck KGaA, Darmstadt, Germany). Anti-GAPDH rabbit polyclonal antibody (1:5000) (Sigma Aldrich, Merck KGaA) was used to normalize the protein content. Horseradish peroxide-conjugated goat anti-mouse or goat anti-rabbit secondary antibody complexes were detected by chemiluminescence (Cell Signaling Technology, Beverly, MA, USA). Imaging and densitometric analyses were performed with a UVITEC Mini HD9 acquisition system (Alliance UVItec Ltd., Cambridge, UK).

### 4.5. Semi-Quantitative RT-PCR

Total RNA was extracted from each cell line using an Aurum Total mini kit (BioRad Laboratories, Milan, Italy). cDNA was synthesized from 1 μg of total RNA using the PrimeScript^TM^ RT Master Mix (Takara Bio Inc., Beijing, China) according to the manufacturer’s instructions. Quantitative real time RT-PCR was performed in a reaction mixture containing SYBR qPCR Master Mix (Vazyme Biotech Co., Ltd., Nanjing, China) and 25 pmol of forward and reverse primers. Reactions were carried out using a CFX96 Real Time System (Bio-Rad Laboratories). All samples were run in triplicate and relative expression was determined by normalizing the results to actin mRNA. Sequences of primers are reported in Supplementary Table S2.

### 4.6. Cytokine Protein Array

The expression of 20 human cytokines was analyzed using a commercially available antibody array system (RayBio^®^ C-Series Human Inflammation Array C1 Map, RayBiotech Life, Inc., Peachtree Corners, GA, USA) that uses membrane-bound cytokine-specific antibodies to capture cytokines in biological fluids. The procedure was performed as per the manufacturer’s instructions. Cells were serum-free seeded in 6 mm culture dishes for 96 h. The cytokine array membranes were blocked in 2 mL 1 × blocking buffer for 30 min at room temperature (RT) and then were incubated with 1 mL of conditioned medium at 4 °C overnight. The medium was then decanted from each container, and the membranes were washed three times with 2 mL 1 × wash buffer I, followed by two washes with 2 mL 1 × wash buffer II at room temperature. Next, the membranes were incubated in biotin-conjugated primary antibodies for 2 h at RT and then washed as described above before incubation in 1:1000-diluted horseradish peroxidase-conjugated streptavidin for 2 h. The membranes were then washed thoroughly and incubated with a chemiluminescent ECL substrate at RT for 5 min. Imaging and densitometric analyses were performed with UVITEC Mini HD9 acquisition system (Alliance UVItec Ltd.).

### 4.7. Scratch Wound Assay

Melanoma cells were plated on 35 mm dishes and allowed to grow until confluence. To obtain a standardized cell-free area, the cell monolayer was then wounded with a pipette tip (1 mL), washed, and maintained in CAF or NHF supernatant diluted 1:1 with culture medium for 24, 48, and 72 h. The cultures were fixed immediately after the scratch (T0), at 24, 48, and 72 h. Images were recorded using a CCD camera (Zeiss, Oberkochen, Germany) and the migratory ability was quantified by measuring the leading edge distance using the Zen 2.6 software (Zeiss). Results are expressed as the percentage of reduction with respect to T0, which was set as 100. For each time point evaluated, the reduction of edge distance of melanoma cells treated with NHF supernatant was compared with that of the cells maintained with CAF supernatant.

### 4.8. Immunofluorescence

Cells seeded on coverslips were fixed with cold methanol for 4 min at −20 °C or with 4% of paraformaldehyde for 20 min at room temperature followed by 0.1% Triton X-100 to allow for permeabilization. Cells were then incubated with the following primary antibodies: anti-αSMA mouse monoclonal antibody (1:400) (Sigma Aldrich, Merck KGaA), anti-vimentin rabbit antibody (1:400) (AbCam, UK), and anti-fibronectin mouse monoclonal antibody (1:100) (Santa Cruz Biotechnology Inc., Santa Cruz, CA, USA). The primary antibodies were visualized using goat anti-rabbit Alexa Fluor 555 conjugate and goat anti-mouse Alexa Fluor 488 conjugate antibodies (1:800) (Cell Signaling Technology). Coverslips were then mounted using ProLong Gold antifade reagent with DAPI (InVitrogen, Life Technologies Corporation, Eugene, OR, USA). The fluorescence signals were analyzed by recording stained images using a CCD camera (Zeiss).

### 4.9. Statistical Analysis

Results in the figures are representative of several experiments performed with at least three to seven cell lines from independent donors. Quantitative data were reported as mean ± standard deviation (SD) calculated by Excel software program. Student’s *t*-test was used to assess statistical significance with thresholds of * *p* ≤ 0.05 and ** *p* ≤ 0.01.

## Data Availability

The datasets used or analyzed during the current study are available from the corresponding author on reasonable request.

## Supplementary Materials

The following are available online at https://www.mdpi.com/article/10.3390/ijms22147255/s1.
