# Supplementary material for: Profiling Cancer-Associated Fibroblasts in Melanoma
Source: Int J Mol Sci. 2021 Jul 6;22(14):7255. doi: 10.3390/ijms22147255 (PMC8306538; doi:10.3390/ijms22147255)
Supplement: Supplementary file 1 [file ijms-22-07255-s001.zip › ijms-1241078-supplementary.pdf]

Suppl Table S1. Features of melanomas

Tissue Sample	Cells	Stage	Age	Anatomic region
Melanoma12	CAF	pT2b	56	Gluteus
Melanoma13	Mel13	pT3b	62	Leg
Melanoma16	Mel16	pT4b	46	Abdomen
Melanoma56	CAF, Mel56	pT4b	48	Shoulder
Melanoma75	CAF	pT4b	89	Shoulder
Melanoma77	CAF, Mel77	pT4b	71	Shoulder
Melanoma85	CAF	pT4b	89	Arm
Melanoma101	CAF	pT4b	82	Arm
Melanoma103	CAF	pT4a	71	Arm
Melanoma104	CAF	pTx	65	Occipital region

Suppl Table S2. Primers for Real Time Quantitative Polymerase Chain Reaction

Target Gene	Foward Primer	Reverse primer
βactin	5'-GACAGGATGCAGAAGGAGATTACT-3'	5'-TGATCCACATCTGCTGGAAGGT-3'
α-SMA	5'-GCAGCCCAGCCAAGCACTG-T-3'	5'-TGGGAGCATCGTCCCCAGCA-3'
COLL11	5'-AGCACAGACGGAGATAAATGGC-3'	5'-CGACAAGCATACCAGGCTCA-3'
CXCL10	5'-AGCAGAGGAACCTCCAGTCT-3'	5'-AGGTACTCCTTGAATGCCACT-3'
CXCL12	5'-GCCCTTCAGATTGTAGCCCG-3'	5'-AGTCCTTTTTGGCTGTTGTGC-3'
b-FGF	5'-CTGGCTTCTAAATGTGTTACGGA-3'	5'-GCCCAGGTCCTGTTTTGGAT-3'
FAP	5'-GGCTTATCACCTGATCGGCAA-3'	5'-ACAAATTCTCCATTGCTAAGGTCA-3'
Fibronectin	5'-CCTCGAAGAGCAAGAGGCAG-3'	GCTTCAGGTTTACTCTCGCA-3'
FSP1	5'-CTTGGTTTGGTGCTTCTGAGAT-3'	5'-TGGACACCATCACATCCAGG-3'
Glut1	5'-TGGCATCAACGCTGTCTTCT-3'	5'-AGCCAATGGTGGCATACACA-3'
Glut3	5'- CGAACTTCCTAGTCGGATTG-3'	5'-AGGAGGCACGACTTAGACAT-3'
Hexo2	5'-CCTCGGTTTCCCAACTCTGC-3'	5'-GGTCAACCTTRCTGCACTTGG-3'
HGF	5'-CGCTGACAATACTATGAATGACAC-3'	5'-GCTCGTGAGGATACTGAGAATC-3'
HMGCoA	5'-TACCACCAATGCCTGCTACG-3'	5'-TGGCATAACGACCATCCCAG-3'
IGF	5'-TCAGCAGTCTTCCAA-3'	5'-CACAGCGCCAGGTAG-3'
IL1α	5'-CGCCAATGACTCAGAGGAAGA-3'	5'-AGGGCGTCATTCAGGATGAA-3'
IL1β	5'-CTGAGCTCGCCAGTGAAATG-3'	5'-TTTAGGGCCATCAGCTTCAAA-3'
IL6	5'-AGCCACTCACCTCTTCAGAACG-3'	5'-GGTTCAGGTTGTTTTCTGCCAG-3'
IL8	5'-CTTGGCAGCCTTCCTGATTTC-3'	5'-TTCTGTGTTGGCGCAGTGTG-3'
MFAP5	5'-TTCCACAGATGACTTGGCCTC-3'	5'-CGATGCACAGAGTAGAGCCTT-3'
MMP1	5'-CTGGCCACAACTGCCAAATG-3'	5'-CTGTCCCTGAACAGCCCAGTACTTA-3'
MMP2	5'-TCTCCTGACATTGACCTTGGC-3'	5'-CAAGGTGCTGGCTGAGTAGATC-3'
MMP9	5'-GACGATGACGAGTTGTGGTCC-3'	5'-GGCCCTCGAAGATGAAGGGG-3'
NKG2D	5'-TCCCTCTCTGAGCAGGAATC-3'	5'-CCACGAATCCACCCCATCAA-3'
PDGFRα	5'-GTGTGGGACATTCATTGCGG-3'	5'-TAGGCTCAGCCCTGTGAGAA-3'
PDGFRβ	5'-GGAGAGGGCAGTAAGGAGGA-3'	5'-GAAGCCGCATGGTGTCCT TG-3'
PDL1	5'-TGCAGGGCATTCCAGAAAGA-3'	5'-ACCGTGACAGTAAATGCGTTC-3'
PDL2	5'-AGGGAAGTGAACAGTGCTATCTG-3'	5'-CTGCAGGCCACCGAATTCTT-3'
PTGIS	5'-TATCCTTTGGCAAGCGGAGC-3'	5'-TCAGCACGCTATCAAGCACA-3'
SCF	5'-AAGAGGATAATGAGATAAGTATGTTG-3'	5'-TTACCAGCCAATGTACGAAAGT3'
TGFβ	5'-GCCCTGGACACCAACTATTG-3'	5'-CGTGTCCAGGCTCCAAATG3'
TIMP2	5'-GTTTATCTACACGGCCCCCT-3'	5'-TCGGCCTTTCCTGCAATGAG-3'
VEGF	5'-GTTGACCTTCCTCCATCC-3'	5'-TTCTCTGCCTCCACAATG-3'
